# The Cost of Routine Follow-Up in Total Joint Arthroplasty and the Influence of These Visits on Treatment Plans

**Published:** 2018-08-30

**Authors:** Thomas J. Hendricks, Alexander C.M. Chong, Robert P. Cusick

**Affiliations:** 1 University of Kansas School of Medicine-Wichita, Department of Orthopaedics, Wichita, KS; 2 Sanford Health, Sanford Orthopedics & Sports Medicine, Fargo, ND; 3 Sanford Health, Sanford Sports Science Institute, Sioux Falls, SD; 4 Kansas Orthopaedic Center, PA., Wichita, KS

**Keywords:** arthroplasty, follow-up care, survey, cost of illness

## Abstract

**Introduction:**

Many physicians recommend annual or biennial visits after total hip and knee arthroplasty (THA and TKA). This study sought to establish the cost of a post-operative visit to both the health care system and patient and identify if these visits altered patient management.

**Methods:**

A prospective cohort study was conducted using patients presenting for follow-up after THA or TKA from April through December 2016. All surgeries were performed by a single orthopaedic surgeon in Wichita, Kansas. All eligible subjects that met the inclusion criteria received and completed a questionnaire about the personal cost of the visit and their assessment of their function and outcome after total joint arthroplasty. The physician also completed a questionnaire that examined the cost of the visit to the health care system and whether the clinical or radiographic findings altered patient management.

**Results:**

Fifty-six patients participated with an average length of follow- up of 4.5 ± 4.1 years since surgery. The average patient cost was $135.20 ± $190.53 (range, $1.65 – $995.88), and the average visit time for the patient was 3.9 ± 2.9 hours. Eighty percent of patients reported no pain during the clinic encounter, and 11% reported loss of function. Eighty-four percent thought the visit was necessary. Physician time for each visit lasted 12.9 ± 3.7 minutes (range, 10 – 20 minutes). Only 9% of patient encounters resulted in an alteration in patient management. This occurred at an average follow-up time of 3.6 ± 1.8 years after the index procedure. The average cost of each visit to the health care system at large was $117.31 ± 60.53 (range, $93.90 – $428.28).

**Conclusions:**

The findings of this study advise total joint patients and orthopaedic surgeons regarding the cost of routine post-operative appointments and whether these visits alter patient management. The majority of the routine follow-up visits after THA and TKA did not result in an alteration in patient management, but added substantial cost to the health care system.

## INTRODUCTION

Total hip (THA) and knee (TKA) arthroplasty are remarkably effective and highly successful surgical treatment options for patients with refractory, end-stage hip and knee arthritis.^1^–^9^ These operations are two of the most common procedures performed by orthopaedic surgeons in the United States. Projections estimate that by the year 2030 the demand for THA will grow 174% to 572,000 per year, while the demand for TKA will grow 673% to 3.48 million procedures annually. 10–11 These numbers are expected to rise as patients live longer and as these procedures are performed more often in younger (55 years of age or less) and more active patients.^12^–^17^

Even though these procedures have shown great success with high patient satisfaction rates,^18^–^22^ arthroplasty failures continue to remain a challenge. Revision THAs and TKAs are costly to the health care system and the patient’s overall well-being.^23^–^25^ Frequently, patients may be asymptomatic before such failure occurs. Timely intervention for patients with asymptomatic complications is beneficial for their long-term health outcomes. Therefore, there is a need for close post-operative monitoring to detect and manage these complications before catastrophic failure arises. Many physicians recommend routine follow-up after total joint procedures.^26^ Such follow-up visits, however, can be costly for both patient and the health care system. Given the overall state of American health care at the present and the anticipated rise in the number of joint replacements performed, it is imperative to find a cost-effective model for managing patients after surgery. One possible source of cost containment includes the timing and frequency of follow-up visits to the orthopaedic surgeon and the routine use of radiographs. Additionally, the cost to the patient is as relevant as those incurred by the system. Elimination of waiting and travel time with the associated costs incurred to the patients may improve their satisfaction with care.^27^–^32^

With the improvements in prosthetic design and materials used in total joint arthroplasty, revision in asymptomatic patients is uncommon within the first seven years post-operatively;^33^ therefore, the need for annual or biennial routine follow-up of these patients after total joint replacement is questioned. To our knowledge, explicit written guidelines or standards for long-term THA/TKA follow-up care do not exist, although some general references are noted in the literature. 28,34,35 Because of the lack of specific guidelines and studies in the U.S. for long-term follow-up, the objective of this study was to assess the totality of these costs to the patient, physician, and health care system, and determine whether these routine post-operative clinic visits alter patient management. By making such determinations, potential sources of cost savings for patients and surgeons could be identified and employed, improving the clinical decision-making processes and enhancing patient satisfaction.

## METHODS

Institutional review board approval was obtained for this prospective cohort study. Subjects were selected from those patients presenting for follow-up after THA or TKA from April through December 2016 at a single institution by a single board-certified orthopaedic surgeon in Wichita, KS. Inclusion criteria consisted of patients who underwent either THA or TKA for treatment of primary osteoarthritis and at least 40 years or older at the time of clinic visit. Exclusion criteria consisted of subjects who were less than one year post-operative from THA or TKA, diagnosis of inflammatory arthritis or post-traumatic osteoarthritis, prior revision of THA or TKA, previous joint sepsis, subjects who were being followed at closer intervals than typical protocol for concern of THA or TKA failure, and subjects whose index arthroplasty was not performed by the lead physician.

Each eligible subject that volunteered was informed about the purpose of the study, and received and signed a consent form upon presentation to the orthopaedic clinic for follow-up. Questionnaires (Appendix A) completed by subjects had questions pertaining to the personal cost of the clinic visit. Issues of interest to the researchers were time elapsed since surgery, whether the subject was experiencing pain or loss of function in their joint, how much time the clinic visit took from their day, how many miles they drove to their appointment, total estimated monetary cost of the visit (includes gas, lost wages, co-pays, etc.), whether they had a friend or relative accompanying them and the cost of this visit to that person, and whether the visit was necessary. The mileage was calculated from the travel distance between the subject’s home and clinic using Google Maps, and the cost of mileage was calculated using the IRS reimbursement rate for mileage driven for medical purposes of $0.235 per mile.

The lead physician completed a questionnaire (Appendix B) for each eligible subject that participated in this study. The physician questionnaire investigated the type of total joint arthroplasty, time spent on the visit, radiographs or laboratory studies ordered, whether the management plan changed because of the visit, and whether the physician felt the visit was necessary. The cost of the visit to the physician and the health care system, including radiographs, laboratory studies, and cost of the outpatient visit for a given level (1 – 5) for an established patient in a non-facility setting, was determined using the Centers for Medicare & Medicaid Services (CMS) 2015 Physician Fee Schedule, using the non-facility cost and Kansas locality (Table 1). Data collection also included subject demographics information such as sex, age, height, weight, body mass index (BMI), surgical procedure type, date of surgery, and the subject’s home address.

Descriptive statistics of the mean, standard deviation, and range were determined using the continuous variables of time elapsed since surgery, subjects’ demographics (age, height, weight, BMI), estimated time of the clinic visit, travel distance, estimated average total cost for the patient, physician’s clinic visit time, and estimated average total cost for the health care system. Data entry was accomplished using Microsoft Excel 2013 (Microsoft, Redmond, WA).

## RESULTS

A total of 58 consecutive subjects participated in the study, of which two were excluded based on the inclusion/exclusion criteria. The population included 27 females (48%) and 29 males (52%; Table 2). The average age was 68 ± 10 years (range, 46 – 86 years) with an average BMI of 33.58 ± 7.73 (range, 15.66 – 52.61). The average length of follow-up since surgery was 4.5 ± 4.1 years (range, 1.0 – 19.3 years). Twenty-one (38%) of the 56 patients had more than one total joint arthroplasty procedure performed. Forty-one (62%) had TKA and 19 (29%) had THA. There were five subjects (8%) with bilateral THA performed and one subject (2%) had bilateral TKA performed.

Overall, the majority of subjects were satisfied with functional outcomes of their total joint arthroplasty. Forty-five (80%) out of the 56 subjects reported no pain during the clinic encounter. This result indicated improvement (46%) or no change (43%) since their last encounter (Table 3). There were a minority of subjects (11%) that reported loss of function in their total joint arthroplasty; however, only three subjects (5%) stated their function had worsened since their last encounter. There were 29 subjects (52%) who reported improvement in function, and 24 subjects (43%) who reported no change in function. Despite the time and cost of each encounter, the majority of subjects (84%) thought that the visit was necessary. There were only nine subjects (16%) that thought the visit was unnecessary. For this subgroup of patients, there were two female subjects and seven male subjects who lived an average of 123.9 ± 172.8 miles (range, 9.4 – 430.0 miles) away from the clinical site, and their average follow-up time was 2.3 ± 1.9 years (range, 1.0 – 5.4 years).

The estimated average cost for the subjects for each encounter was $135.20 ± $190.53 (range, $1.65 – $995.88). Each visit, including travel time, required 3.9 ± 2.9 hours (range, 0.5 – 12.0) of the subjects’ time. The distance traveled for each patient varied considerably, resulting in an average travel distance of 131.2 ± 158.5 miles (range, 2.6 – 580.0 miles). There were 29 subjects (52%) who came with a companion to the encounter.

From the physician’s perspective, each visit lasted approximately 12.9 ± 3.7 minutes (range, 10 – 20 minutes; Table 3). Out of the 56 subjects, only five (9%) encounters resulted in an alteration in patient management beyond routine follow-up. This occurred an average of 3.6 ± 1.8 years (range, 1.1 – 5.3 years) after the index procedure. One patient received a three-phase bone scan to rule out aseptic loosening. Another patient complained of radiculopathy, and a magnetic resonance imaging (MRI) study of the lumbar spine was ordered. A third patient demonstrated clinically significant quadriceps weakness, and a lower extremity electromyogram and nerve conduction velocity study was ordered. A fourth patient complained of symptomatic patellar osteophytes and global instability. This patient was scheduled for surgery, consisting of open osteophyte excision and polyethylene liner exchange. The final patient had a laboratory panel ordered to rule out periprosthetic infection. For these five subjects, the estimated average cost was $155.16 ± $239.18 (range, $6.96 – $577.29) while the cost of these appointments to the health care system was $261.78 ± $145.60 (range, $100.00 – $428.28), and the average cost of each visit to the health care system at large was $117.31 ± $60.53 (range, $93.90 – $428.28).

## DISCUSSION

This study accurately delineated the costs of each clinical encounter from a patient and system perspective using prospectively gathered data from patient and physician questionnaires. The majority of patients were satisfied with the pain and function of their total joint arthroplasty, which is in line with reported data.^36^ This study accurately assessed patients’ perceptions of the visit in real time instead of relying on patient recall during post-visit follow-up phone calls, and limiting any recall bias. This study assessed whether the encounter resulted in any change in management above and beyond routine follow-up.

Surveys and questionnaires as a method for developing information about clinical practice have been used in many medical specialties. 37,38 Most patients in this study reported by questionnaire they were satisfied with their total joint from a pain and functional standpoint. The majority (91%) of these follow-up visits were not viewed as necessary by the lead surgeon and did not result in an alteration in patient management at an average of 4.5 ± 4.1 years (range 1.0 – 19.3 years) after surgery. These findings contradicted the results of prior studies. Teeny et al.^26^ surveyed 682 members of the American Association of Hip and Knee Surgeons and found that the majority (80%) of respondents favored annual or biennial visits for uncomplicated total hip and knee arthroplasties. Furthermore, their study showed an agreement among members that more frequent follow-up may be necessary in the setting previously identified signs of early failure, previous joint sepsis, previous revision surgery, and poor bone quality.

Many physicians recommended scheduling routine post-operative follow-up appointments after primary total joint arthroplasty, even if patients are asymptomatic. The objective of routine outpatient assessment of asymptomatic patients is to evaluate and detect early signs of failure and to guide recommendations for early intervention. Some issues that factor into this decision-making process include implant design, materials, manufacturing methods, implant fixation methods, surgical technique, implant shelf life, presence of bone grafts,^39^–^43^ patient young age,^44^–^46^ activity level,^47^,^48^ patient’s weight,^49^ revision arthroplasty,^50^–^52^ poor bone quality,^53^ history of joint sepsis,^54^–^56^ compromised immune status, and other underlying disease processes.^45^,^47^ The initial signs of failure detected may be bone loss secondary to osteolysis, resulting in more complex revision procedures with higher risks, higher costs, and less successful outcomes. Some early signs of failed total joint arthroplasty include an increase in pain or a decrease in joint function. Persistent pain and swelling may indicate implant-loosening, wear, or infection; the decline in joint function may cause a limp, stiffness, or instability. Patients who demonstrate these symptoms and signs may require revision joint arthroplasty.

Christensen and Folkmar^57^ performed a retrospective chart review study in Denmark to examine whether radiographs at three and twelve-month marks post-operatively resulted in any change in primary elective cementless THA patient management. Their results showed that at three months, only eight (4%) of 216 cases showed any subsidence (all cases were <10 mm), and only one out of the eight patients was treated with crutches while the others received closer follow-up. At 12 months, only two patients (1%) showed stress shielding and were given further follow-up. They concluded that routine radiographs in that first year did not offer any benefit and would only be warranted when the patient presented with a specific complaint regarding their total joint. Hacking et al.^33^ performed a prospective analysis of 110 THAs over a four-year period, and they found that only four (3.6%) of the 110 cases were for asymptomatic revisions in the first seven years after primary THA. Other studies supported “no follow-up” until several years after primary THA.^58^,^59^ The findings in the present study corroborated these results. In the present study, all patients received a clinical and radiographic examination during their encounter, but rarely (9%) did this lead to an alteration in care. It is no doubt that detection of silent but potentially significant problems in total joint arthroplasty may be enhanced by regular, periodic follow-up, which would allow the impending failure to be detected at an earlier stage, thus reduce the increasing health care costs and burdens associated with revision THA and TKA. The current practice of routine follow-up of asymptomatic THA or TKA, however, may be excessive, costly, and unnecessary, and a less resource-intensive review method may be more appropriate.

Interestingly, our results indicated that 84% of total joint arthroplasty patients preferred routine follow-up. This result contradicted reports from a study performed by Sethuraman et al,^28^ which looked at 100 asymptomatic or minimally symptomatic total joint arthroplasty patients with two or more years prior between June 1998 and October 1998. Their results showed that nearly half of their patients preferred to avoid the routine follow-up secondary to lost time and wages, and patient-provider telephone care was preferred. One possible explanation for this disparity is due to pre-operative patient education. Pre-operatively, most patients are informed, either by their surgeon or the internet, that they will need routine annual or biennial follow-up to ensure they are not developing any post-operative complications. Many of these patients, including those without symptoms, may feel these visits are crucial in preventing catastrophic problems with their total joint. Educating patients regarding early signs and symptoms of total joint arthroplasty failure is crucial if physicians plan to eliminate early routine follow-up visits in the first few years after surgery when patients are less likely to develop these complications. Patient education, such as the benefit of proper diet, acceptable levels of post-arthroplasty exercise, and smoking cessation, should be emphasized. Tobacco has been shown to hinder bone healing. Alcohol consumption (beer, liquor, or wine) of three or more units per day will have consequential effects on bone health, leading to lower bone mineral density when compared with more moderate drinking.^60^ Education on avoidance of preventable falls also has a major impact on reducing further periprosthetic and fragility fractures, especially in patients with osteoporosis who often experience muscle weakness, postural deformity, and poor balance.^61^ Patients who undergo tailored exercises and intervention have a decrease in fall rate in the community. 62 These measures with appropriate patient education could reduce the need for routine early follow-up after a total joint arthroplasty.

The cost to the patient is as relevant as those incurred by the health care system. Elimination of waiting and travel time with the associated costs incurred to the patients may improve their satisfaction with care.^29^–^32^ Sethuraman et al.^28^ reported their patient population could have saved wages averaging $135 for each clinic visit in Philadelphia, PA in 1998. This study also determined a similar average cost to the patient. In the present study, the average cost was $135.20 ± $190.53 (range, $1.65 – $995.88). In the Midwest, such variation is not unexpected when one considers the geographical area orthopaedic surgeons may serve.

When factoring in the driving time and distance along with lost wages, it makes it easier to recognize how such a visit may prove more expensive for a patient living further away than for another residing in the same zip code. For this reason, telemedicine may become an option for the future. Patients could have x-rays taken at their local hospital or primary care provider’s office and have the imaging sent to their surgeon for review, followed by a telemedicine encounter to discuss how the patient’s total joint is performing. However, telemedicine is only a virtual interaction. The encounter would be missing the physical examination component, which is an important part of the evaluation process. Without it, there exists the possibility that certain issues could be missed. To our knowledge, no studies have been performed comparing the efficacy of telemedicine interactions compared to traditional patient encounters concerning detection of complications after total hip or knee arthroplasty. Such research, however, could represent an area of future study for follow-up of total hip and knee arthroplasty patients.

As health care costs increase, more emphasis has been placed on cost containment. The numbers generated in our study represent one possible source of savings to the health care system. Bolz et al.^27^ used a decision-analytic Markov model to compare the costs and health outcomes of three follow-up strategies after primary total joint arthroplasty and demonstrated that without routine follow-up for the first seven years after surgery, there would be a total savings to the system of $11.9 million and gains of between 1.8 to 8.8 quality-adjusted life years for patients. However, it is important to temper the goal of cost savings by balancing with it a need to provide satisfactory patient care. Identifying pre-operatively which patients may wish to have routine post-operative visits and benefit from them versus those that would prefer less frequent follow-up intervals, may allow for a strategy in which health care costs are decreased while concomitantly increasing patient satisfaction.

Several concerns may be raised regarding the validity of our model and applying our results to the management of routine follow-up visits after total joint procedures. The most significant limitation in this study was small sample size, and the patients were drawn only from one local physician. This prevented application of tests of significance due to insufficient power. The low number of subjects participating was unavoidable because the office staff of the lead physician experienced a high turnover rate during the data collection period. Repeating this study with a larger number of enrollees would be beneficial, thus making the data more reliable for a treatment analysis. This would be beneficial to practitioners deciding on how to manage post-operative follow-up after total joint arthroplasty. Another limitation was that the physician reviewing the prospective group was not blinded to the purpose of the study. This situation could introduce a collection bias that might underestimate the importance of routine post-operative follow-up. Another weakness was that the cost determinations were only estimates based on Medicare reimbursement rates for billed CPT codes. The present study did not collect data regarding patients’ insurance policies. It was likely that several patients had insurance other than Medicare that paid for their health care. Our estimated costs may not reflect the true amount the facility billed, nor what was paid by the insurer. The cost to the patient was only an estimate based on patients’ travel distance to and from the clinic and potential lost wages of both themselves and their companions.

Additionally, this study identified a discrepancy between patients and the physician regarding the usefulness of each post-operative visit. However, the questionnaires did not explore the rationale behind these beliefs. Examining these thoughts could identify the etiology behind the difference and provide physicians with a better understanding of their patients’ psyches, thus improving the doctor-patient relationship. Despite these limitations, our data were valid. Further research using a larger study population with multiple surgeons and employing a cost-effectiveness model is needed, and subjects should be followed prospectively at each post-operative visit to support and expand upon our findings further.

## CONCLUSION

In summary, this study illustrated that the majority of post-operative follow-up visits, especially for those asymptomatic patients, did not result in an alteration in patient management, but added substantial cost to the health-care system. Future studies are needed to determine, fully and accurately, the cost-effectiveness of these visits and how many patients must be seen routinely to prevent total joint failure.

## Figures and Tables

**Table 1:** Cost data used in current study.

Code	Description	Cost (USD)*
99211	Level 1 Outpatient Visit, Established Patient	18.62
99212	Level 2 Outpatient Visit, Established Patient	41.03
99213	Level 3 Outpatient Visit, Established Patient	68.71
99214	Level 4 Outpatient Visit, Established Patient	102.17
99215	Level 5 Outpatient Visit, Established Patient	137.99
73510	Radiograph of the hip, unilateral, 2 views	34.05
73520	Radiograph of the hip, bilateral, 2 views	36.40
72170	Radiograph of the pelvis, anterior posterior	25.19
73560	Radiograph of the knee, 1 or 2 views	26.81
73562	Radiograph of the knee, 3 views	31.36
72564	Radiograph of the knee, 4 views	36.58
73565	Radiograph of the knees, bilateral, anterior posterior, weight bearing	30.03
78315	Bone scan, 3 phase	328.21
85025	Complete blood count with differential	10.58
85652	Erythrocyte sedimentation rate, automated	3.68
86140	C-reactive protein	4.70

* CMS/Medicare 2015 data, Kansas locality, non-facility price.

**Table 2:** Patient demographics.

Total number of patients	Female	27 (48%)
	Male	29 (52%)
Mean age (years)		68 ± 10 (range, 46 – 86)
Height (inches)		66 ± 4 (range, 59 – 74)
Weight (lbs.)		209 ± 52 (range, 97 – 346)
BMI (kg/m^2^)		33.58 ± 7.73 (range, 15.66 – 52.61)
Follow-up time (years)		4.5 ± 4.1 (range, 1.0 – 19.3)
Number of patients with >1 joint replacement		21 (38%)
Joint type	Hip	19 (29%)
	Knee	41 (62%)
	Bilateral Hip	5 (8%)
	Bilateral Knee	1 (2%)

**Table 3:** Summary results.

**Patient**	Feel pain at vist	Yes	11 (20%)
		No	45 (80%)
	Compare pain vs. previous visit	Improved	26 (46%)
		Worse	6 (11%)
		No change	24 (43%)
	Loss function	Yes	6 (11%)
		No	50 (89%)
	Function vs. previous visit	Improved	29 (52%)
		Worse	3 (5%)
		No change	24 (43%)
	Visit necessary?	Yes	47 (84%)
		No	9 (16%)
	Family member accompany?	Yes	29 (52%)
		No	27 (48%)
	Estimated time taken (hour)		3.9 ± 2.9 (range, 0.5 – 12.0)
	Travel distance (mile)		131.2 ± 158.5 (range, 2.6 – 580.0)
	Estimated average total cost for patient		$135.20 ± $190.53 (range, $165 – $995.88)
**Surgeon**	Office visit time (minutes)		12.9 ± 3.7 (range, 10 – 20)
	Alter management plan	Yes	5 (9%)
		No	51 (91%)
	Visit necessary?	Yes	5 (9%)
		No	51 (91%)
	Estimated average total cost for health care system		$117.31 ± $60.53 (range, $93.90 – $428.28)
